# Research on Silver-Based Wound Dressing: An Ontological Analysis

**DOI:** 10.3390/antibiotics15050462

**Published:** 2026-05-02

**Authors:** Prabir K. Dutta, Thant Syn, Arkalgud Ramaprasad

**Affiliations:** 1 Department of Chemistry and Biochemistry, The Ohio State University, Columbus, OH 43210, USA; 2 Department of Information Systems, Analytics & Supply Chain, Florida Gulf Coast University, Fort Myers, FL 33965, USA; tsyn@fgcu.edu; 3 Department of Information and Decision Sciences, University of Illinois Chicago, Chicago, IL 60607, USA; prasad@uic.edu

**Keywords:** ontology, neural network, artificial intelligence (AI), silver in health and medicine, chronic wounds, regulations, research history, research roadmap, research pathways, systematic review, systemic review

## Abstract

Background/Objectives: Silver’s ability to kill pathogenic bacteria is being widely researched in environment, consumer, and health-related applications. One topic of voluminous research is the antimicrobial properties of silver and silver in wound dressings. This research literature has been reviewed in articles using qualitative analyses, meta-analyses, systematic reviews, bibliometric analyses, and other grounded methods. We present a new strategy for the analysis of the population of articles on the subject based on an ontology of this topic. Methods: A search of the Scopus database for all peer-reviewed articles on silver in wound dressings yielded a population of 4711 relevant ones. The ontology is a logical deconstruction of the problem: “use of **silver** species on **nanosupports** deposited on a **matrix** with **antimicrobial effectiveness** assayed by **methods** to promote **wound healing** of chronic **wounds** as determined by **recovery**”. Each bolded term denotes a dimension of the ontology, and each dimension denotes a taxonomy of constituent elements. A Convolutional Neural Network (CNN) was trained using a manually mapped subset of articles. The CNN was then used to map the population of articles. Results: Out of the 4711 articles, 3079 dealt with silver and wound dressings; the others involved silver, but were not related to wound dressings and were not considered. Overall analysis shows that three classes of silver encompass the entire field: silver nanoparticles (AgNP) (78% of papers), inorganic silver-ion-containing species (7%) and silver associated with organic molecules (15%). AgNP papers have grown exponentially beginning in the early 2000s; there is no clear trend regarding inorganic silver-containing-species papers; whereas with the silver-organics species papers, there has been growth in the past decades, but now the number of publications is stabilizing. Research on the AgNPs has primarily focused on in vitro testing (54%), with very limited animal testing (17%) and human testing (3%). On the other hand, with silver-organics, animal (30%) and human testing (38%) are prominent. Inorganic silver ion species also have been human-tested extensively (43%). Thus, in clinical applications of silver wound dressings, AgNP lags considerably as compared to the other silver species, though academic research in AgNP is robust. Conclusions: From detailed temporal visualizations of the ontological mapping, the antecedents and consequences of silver in wound dressings are presented. This first ontological analysis is a novel way of visualizing an entire research field and the temporal characteristics of the various dimensions of the ontology provide information on the current state of research as well as where the field is headed.

## 1. Introduction

Medicinal properties of silver have been known for centuries [1]; the earliest reports of use of silver date back to 5000 years ago, as evidenced by archeological discoveries of a silver canister dating back to 1400 B.C. Silver-based wound dressings were used during the First World War (1914–1918), though the exact mechanism of action was unknown [2]. In the early part of the 20th century, silver was used for the treatment of various diseases, such as syphilis [3]. Until the advent of antibiotics, silver enjoyed use as a general-purpose antimicrobial. Now with the advent of antibiotic resistance, silver is enjoying a renaissance. The mechanism of silver as an antimicrobial stems from the coordinating ability of silver to bind to oxygen-, sulfur-, nitrogen- and phosphorus-containing ligands [4,5,6,7]. Since all living biological entities contain these elements, silver ions can bind to lipids, DNA, RNA, proteins and enzymes and interfere with their activity, thereby manifesting an antagonistic role towards the living species. Silver is known to kill both Gram-negative (e.g., *Acinetobacter*, *Escherichia*, *Pseudomonas*, *Salmonella and Vibrio*) and Gram-positive bacteria (e.g., *Bacillus*, *Clostridium*, *Enterococcus*, *Listeria*, *Staphylococcus and Streptococcus*) [7,8,9]. Oxidative stress and metabolic disruption due to silver have been noted in its antimicrobial mechanism [10,11,12]. Silver’s attack on the cell is multi-pronged, and therefore resistance towards silver is difficult for the bacteria to develop, though there have been a few reports of resistance to silver [13,14,15,16]. Disruption of cell membrane activity, including phosphate and sodium ion uptake inhibition, disruption of the proton gradient, and uncoupling of ATP-dependent processes, as well as DNA aggregation have been noted [7,17,18,19,20]. The flip side of such activity is that silver will also bind to functioning biological entities necessary for the function of the organism and thereby cause cytotoxicity [21,22]. Argyria risk, which involves a cosmetic effect of bluish skin increases with high levels of silver [23]. So, the amount of silver used in a specific antimicrobial application is important, killing the bacteria but not injuring the host organism. Pathogenic bacteria can colonize and survive in very diverse environments, including living beings and different surfaces [24]. In living beings, bacteria can cause infection. By surviving on surfaces, bacteria can be transmitted via touch and contaminate food. Bacteria can survive and form deposits in pipes, and maintain colonies in water bodies [25]. Therefore, silver continues to be investigated as an antimicrobial in medical, consumer and environmental applications [26]. Examples include its use in swimming pools [27], silver-impregnated water filters [28], silver-containing textiles [29], and silver use in dental materials [30]. In medical applications, silver-coated catheters [31] and silver bandages are used [26].

This review article focuses on a very specific application of silver antimicrobials in wound dressings for chronic wounds [32]. Wounds exposed to the environment can be readily infected by bacteria, causing issues with wound healing. The presence of silver in wound dressings can kill the bacteria, minimize infection and assist in wound healing. There are many commercial silver wound dressings in use today. One of the first silver species that was used for minimizing infections in burn wounds was silver sulfadiazine [33]. Numerous other silver species have since been introduced in wound dressings, which remains an area of intense research activity. Different forms of silver and supports that promote wound healing are an important area of clinical research. This research activity has led to numerous research articles and patents; thus it is not surprising that there are many review articles in this area. Such review articles in the area of silver and wound healing have included qualitative analyses [34], meta-analyses of randomized control trials [35], systematic reviews of specific features of silver in commercial wound dressings [36], reviews of clinical studies [37] and bibliometric analyses [38]. So, the question that arises is, why another review article?

The approach we have taken in this study is distinct from previous review articles in this area and provides novel features. Our approach is based on an ontological analysis of the entire field, as captured by an exhaustive search involving silver and wound healing. By focusing on the title, abstract and keywords of the manuscripts, we ensure that the search net is cast wide. We take the entire search results and categorize them into an ontological map. The design of the ontology is critical since it provides the basis for the classification of the entire published research into possible classes, as specified by the dimensions and taxonomies of the ontology. Since the number of papers is large, we trained a neural network to do the classification. Such a classification is then mapped onto the ontology, and the map describes the overall state of the field. Because the ontology describes the entire field, there are numerous ways to interpret the findings. One could choose to examine the nature of basic research, or particular methodologies or types of samples. We have decided in this review to use the ontological map to examine the practical/clinical aspects of silver used in wound dressings, and where the field may be headed. The rationale for and the method of ontological analysis are based on the research using it in information systems, healthcare, and project management [39,40,41,42,43], and other fields.

## 2. Ontology of Silver-Based Nanosupport Wound Dressing

To capture the entire field of silver in wound dressings, we developed an ontology of: “use of **silver** species on **nanosupports** deposited on a **matrix** with **antimicrobial effectiveness** assayed by **methods** to promote **wound healing** of chronic wounds as determined by **recovery**”.

An ontology is a novel visualization of a problem in natural language. By organizing the keywords and phrases as a matrix, it represents the combinatorial complexity of a problem concisely, clearly, and comprehensively. The matrix encapsulates the core logic of the problem that can be read by novices and experts. The encapsulated logic serves as a lens to synthesize the discourses about the problem and to generate novel discourses about it. It is a simple visualization of the problem complexity.

The design of silver-based dressings for chronic wounds is a multidisciplinary, combinatorially complex problem. It is based on the integration of research on silver and chronic wound healing from (a) chemistry and chemical engineering, (b) materials science and engineering, (c) biomedicine and pharmaceutical sciences, (d) microbiology and toxicology, (e) clinical medicine, and (f) healthcare management. The design requires discovering an efficacious, effective, and efficient alternative from a very large number of possibilities. The potential market for such dressings is predicted to be large, based on advances in research on the antibacterial properties of silver. However, there is no systemic framework to guide the design. The ontology of silver-based wound dressing is a systemic framework to fill this gap and aid the systematic design of the dressings.

**Figure 1:**
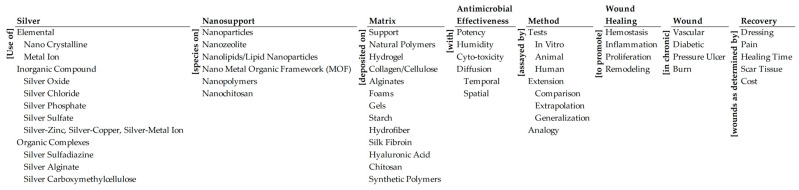
Ontology of silver-based wound dressings.

The proposed ontology below (Figure 1) is a multidimensional, multilayered, and modular framework of the problem based on the foundational disciplines. Its eight dimensions, each shown as a column, represent a key property of a wound dressing. The dimensions are: (a) the silver species that is the active ingredient in the dressing, (b) the nanosupport used for the silver species, (c) the matrix used to deliver the silver species, (d) the antimicrobial effectiveness of the combination of the silver species, nanosupport, and matrix, (e) the wound healing properties, (f) the type of wound, and (g) the recovery requirements. The dimensions include the biomedical, mechanistic, and clinical aspects of wound healing. Each dimension denotes a taxonomy of constituent elements for design, based on research in the foundation disciplines. The dimensions denote the invariant properties of the problem that must be included in design. The elements denote the potential dimensional values in the design; they are the keywords of the problem. Section S1 of the Supplementary Materials defines in more detail the justification of the parts (taxonomy) of each of the dimensions. The framework can be applied at different levels of granularity. The dimensions, the taxonomies, and the levels of the taxonomies can be changed to adjust the scope, size, and scale of the design. The ontology is modular.

In deciding the taxonomies of silver, nanosupport, matrix, and antimicrobial effectiveness we have tried to cover all relevant areas of silver in wound dressings and chronic wound treatments. There are 54 elements chosen because of their importance and relevance; all of them are equally visible. Similarly, all dimensions are equally visible. Subsequent analysis will reveal the relative attention paid to the elements and the dimensions in the research literature. It is possible that one could choose a different set of elements and dimensions. That would give a different perspective on the problem. This is the power of ontology; in a sense the ontology is a living codification of the problem.

It is entirely possible that we may have missed elements. For example, it would be perfectly reasonable to include films under matrix. The reason for not including films is that wound dressings are usually discussed as supports, hydrogels, foams, chitosan, etc. The authors have not seen any literature on silver incorporated in films used as a wound dressing matrix. Usually, films are better defined as the physical form of the matrix. For example, alginate films would fall under alginate, which is an element of the matrix taxonomy.

Similarly, the ontology does not include the mechanistic dimension of silver action. It is a deliberate choice to focus on wound healing and the clinical impact of dressings. If all potential dimensions were included, the review would be exceedingly long. Consequently, dimensions like the mechanisms of silver action are included in the review but are not positioned centrally.

Drawing upon the allegory of the ‘Five Blind Men [sic] and the Elephant’, the ontology is the wise man’s [sic] holistic multidisciplinary formulation of the problem, as opposed to siloed disciplinary formulations. It makes the design ‘elephant’ visible [43]. The columns (dimensions) and taxonomies (layers) of the problem are organized as an ontology with adjacent connecting words/phrases to concatenate all possible combinations of the eight-dimensional problem space in structured natural-English. Each sentence describes a possible pathway to designing silver-based dressings for chronic wounds based on an element from each dimension. It is potentially a key part of the narrative of the design. The ontology provides a top-down view of the design problem, from the big picture to the detail, from the macro to the micro.

There is a very large number of potential design pathways encapsulated by the ontology, as one might expect in a combinatorially complex problem. These pathways can be examined intuitively based on the past knowledge of the researcher, heuristically based on expert knowledge of similar or parallel cases, theoretically based on domain research, and empirically based on laboratory and clinical tests. Sometimes, a subset of the eight dimensions may be considered, with a focus on a segment of a pathway instead of the complete one. The permutations and combinations of elements across the eight dimensions that can be used in the design are numerous. The ontology will help visualize the permutations and combinations and allow the design to proceed systematically. Instead of analyzing the complex combinations ad hoc or incrementally, the ontology will help study them methodically and synoptically.

Consider the following numerical analysis of the complexity of the silver-based wound design problem. There are 1,040,465,790 (54!/(8! × 46!)) possible eight-element combinations of the 54 elements in the ontology. A few of these combinations are semantically meaningful, and most are meaningless. The grouping of 54 elements into the eight-dimensions of the ontology yields 2,184,000 (10 × 6 × 13 × 5 × 7 × 4 × 4 × 5) combinations. These combinations (in conjunction with the connecting words/phrases) are semantically meaningful, potential pathways to address the problem. The clinical value of a pathway must be assessed empirically by the methods listed in the ontology. Using an analogy, the ontology is a ‘Google Map’ to analyze, design, and regulate the design of silver-based dressings for chronic wounds systemically and systematically through feedback and learning. The very large number of possible pathways can overwhelm the bounded rationality [44] of the designers. Instead of muddling through [45] the alternatives, the ontology will help them navigate methodically with feedback [46] and learning, while minimizing potential cognitive biases [47]. The following analysis will highlight the elements and pathways that have been researched and their effectiveness. It will help reduce the complexity to clinically meaningful combinations.

## 3. Methodology

**Figure 2:**
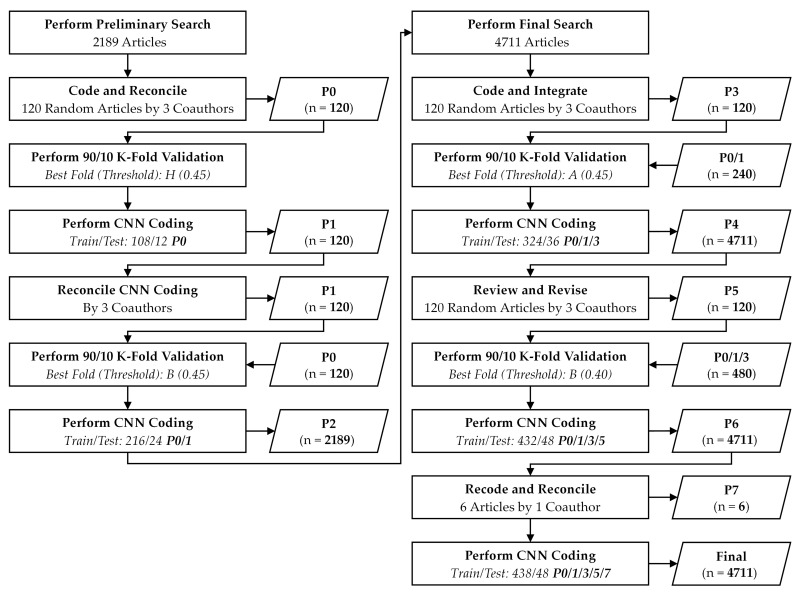
Process of mapping articles to ontology.

All available peer-reviewed articles on silver-based wound dressing were collected from one of the largest databases of scholarly articles, Scopus. The corpus of articles was subsequently mapped to the ontology of silver-based wound dressing. The preliminary search process is described in detail in Section S2.1 of the Supplementary Materials and the PRISMA diagram is shown in Figure S1. The final search expanded the dataset to 4711 articles as described in Section S2.3 of the Supplementary Materials and the PRISMA diagram in Figure S2. The process of mapping articles collected in the preliminary and final searches to the ontology is described in Section S2.2 of the Supplementary Materials and illustrated in Figure 2 below. The final dataset used in the subsequent analysis contained the ontological mappings of 4711 articles.

Mapping the corpus to the ontology based on the large volume of texts in titles, abstracts, and optionally keywords is a resource-intensive process. Moreover, the larger the size of the corpus, the more time-consuming and resource-intensive the mapping process is. Hence, a machine learning technique commonly used in text classification known as a Convolutional Neural Network (CNN) was employed to map the large corpus of articles to the ontology. The CNN is based on a multilayer Neural Network (NN) architecture associated with deep learning. During the early stages of development, deep learning was mainly applied to handwriting recognition [48,49]. Later on, it found success in natural language processing such as sentence recognition [50,51]. In natural language text classification, deep learning techniques are often proven to be more effective and efficient than conventional machine learning techniques [52,53]. For instance, an evaluation of various CNN architectures against other text classification techniques such as logistic regression on top of paragraph vectors and Naïve/Multinomial Bayes Support Vector Machine (SVM) revealed that CNN models in general perform comparably to or better than more established techniques in many test cases [51]. Hence, the CNN was considered a viable and proven method to perform the larger share of coding in this study. The coding process is depicted in Figure 2.

CNN models were trained using ConText software package [54,55]. ConText provides a ready-made environment with a complete set of tools to prepare the vocabulary, train and test CNN models, and perform predictions of coding. The algorithm and hyperparameters used in all CNN models in the mapping process are derived from ConText’s packaged code for multi-label text classification. The important hyperparameters for CNN models including patch size and stride, numbers of layers and size, loss function, and pooling algorithm were fine-tuned over multiple iterations. The bag-of-words model was found to perform better than one-hot and bag-of-n-gram models for the vectorization of region or word embedding. A single max pooling layer using the square loss function over 2000 epochs returned the optimal performance. The performance was evaluated using measures for evaluating regular classification models such as Accuracy, Precision, and Sensitivity, as well as those for multi-class classification models such as F1 Score, Matthews Correlation Coefficient (MCC), Informedness, and Markedness [56,57]. The F1 Score and MCC were found to capture the best performance that balances accuracy, precision, specificity, sensitivity, and used for benchmarking and comparing models in this study.

The primary analysis contained a set of monad maps depicting the number of articles mapped to each ontological element. The number of articles published over time was also analyzed to identify research trends.

**Figure 3:**
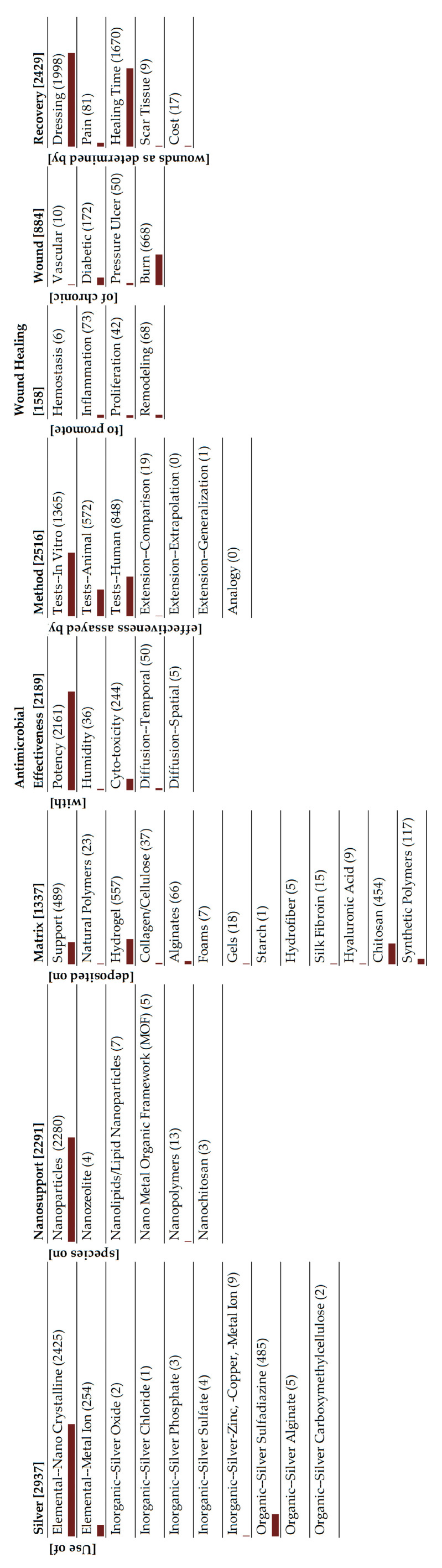
Monad map of the entire corpus.

On the important issue of counting, one may think of what we are doing as classification. For example, an alginate hydrogel would be counted under both these classifications (alginate and hydrogel), though our classification would not distinguish an alginate hydrogel from a PVA hydrogel; both would show up under hydrogels. We are pursuing a classification scheme of the entire literature corpus (>4000 articles) into 54 segments. So, a look at the monad map which is the visualization of the ontology (Figure 3) immediately tells the reader that a certain fraction of the papers have examined “alginates” and “hydrogels”, but it tells us nothing about whether the research is on alginate hydrogels.

The ontology is a visualization of a complex field, made possible by the choice of elements (as detailed now in the Supplementary Materials as to why we chose these 54 elements and provides detailed instructions on how to build an ontology). It is entirely possible that if a reviewer were building an ontology, a different set of elements than the 54 we have chosen could be the output. So, it is subjective in this sense, but if we missed films as an important part of the ontology, we (or anyone else) could redo the ontology with this new field. So, in some sense, the ontology is a living document.

## 4. Results

Figure 3 shows the monad map of the entire set of publications. The map shows the frequency of occurrence of: (a) elements of a dimension in the corpus and (b) each element. The numbers are shown in parentheses adjacent to the dimension and element labels. The bar below each element visually shows the relative frequency of occurrence of that element compared to the maximum frequency of occurrence in the monad map. This monad map figure is central to the ontological analysis because inspection of this figure immediately shows which areas of research are heavily studied, and which are not, for the entire area of silver in wound dressings. We interpret this figure in some detail and derive the current state of the field and where it is headed.

**Figure 4:**
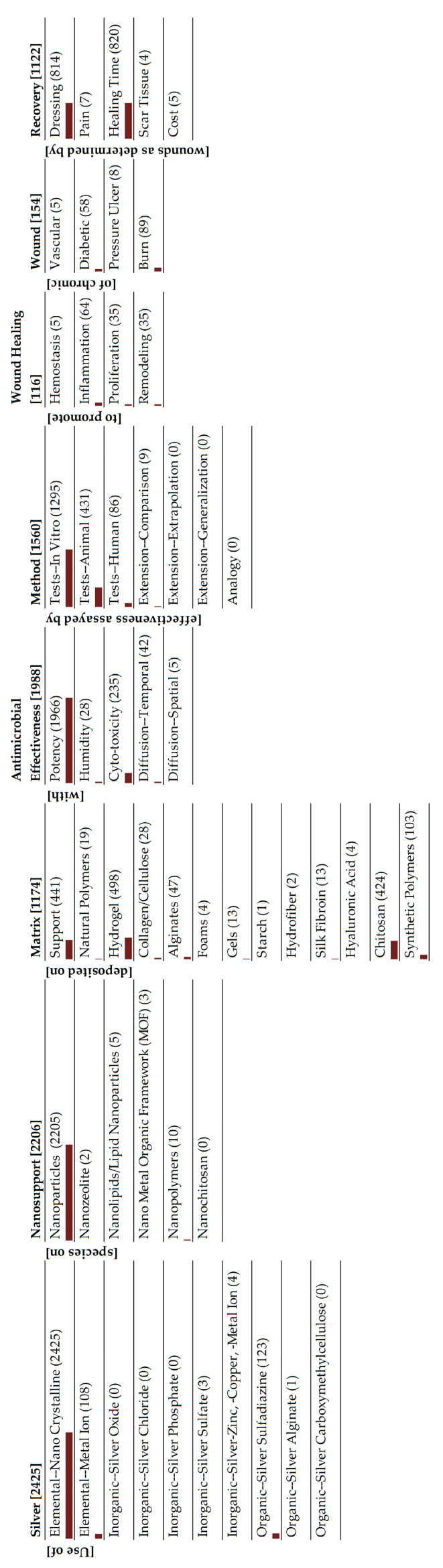
Monad map of metallic silver.

**Figure 5:**
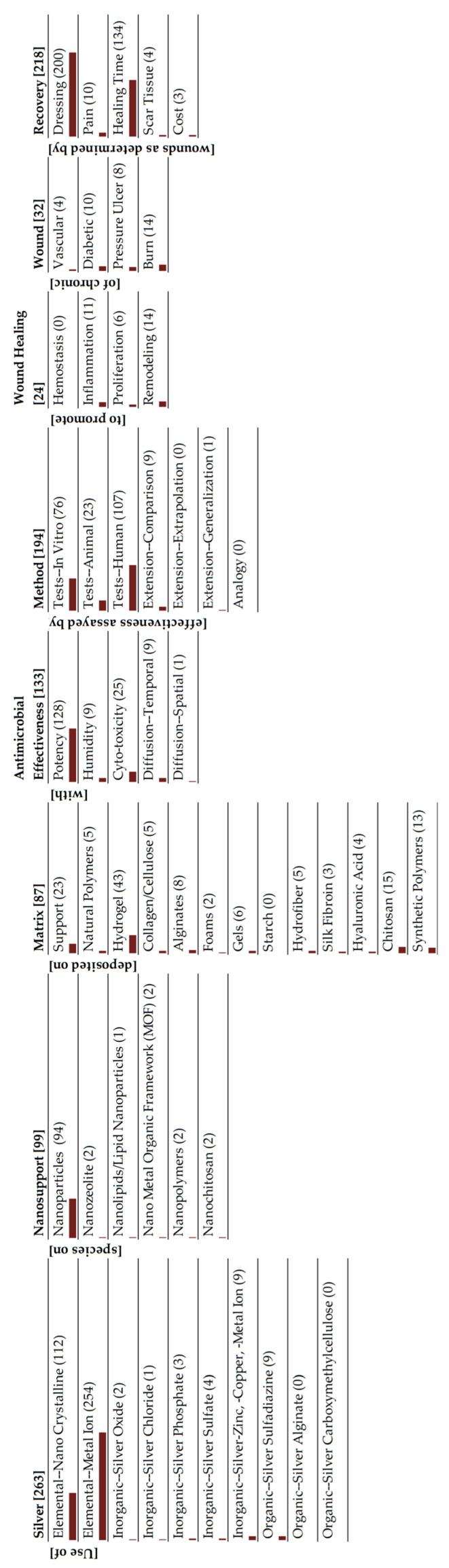
Monad map of inorganic silver species.

**Figure 6:**
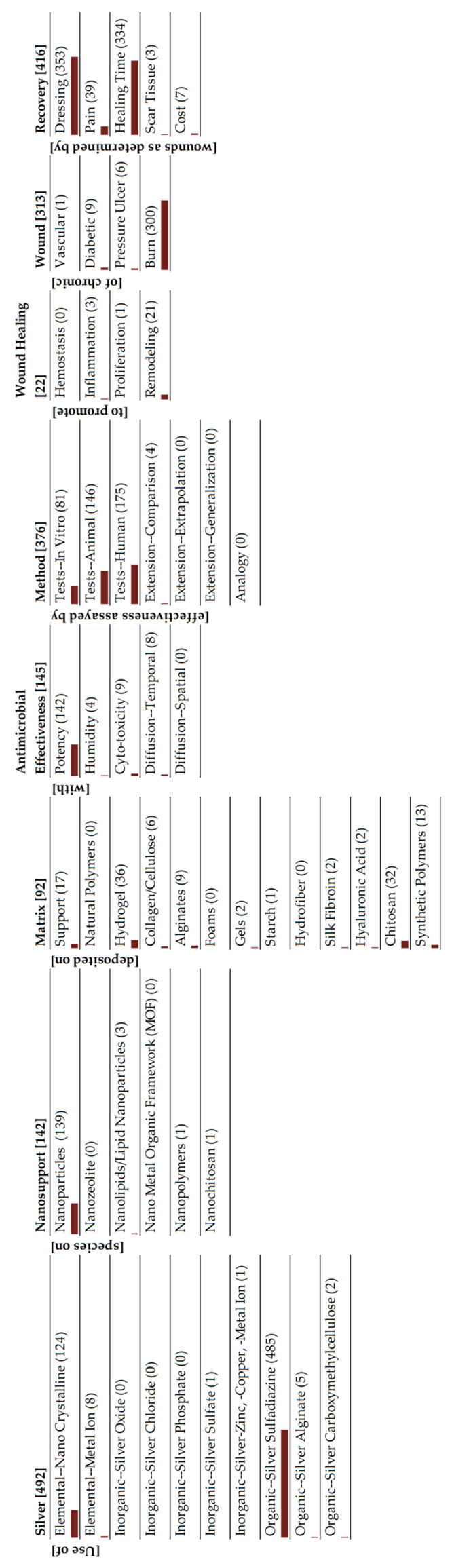
Monad map of organic silver species.

**Figure 7:**
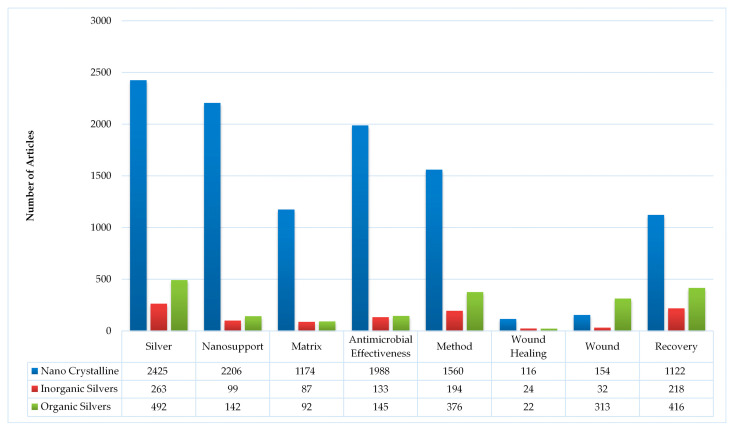
An alternate, simpler visualization of the entire monad map in Figure 3.

There are many ways of interpreting the monad map, depending on the set of information that one is attempting to extract from the entire collection of publications. Since our goal in this review is to understand the role of silver in wound dressings, we will use the classification of silver as our central theme. There are three broad classes of silver species that are being studied: metallic silver, inorganic species and silver complexed to organic moieties. Monad maps for the three classes are readily obtained from the parent map, and are presented in Figure 4, Figure 5 and Figure 6. An alternative summary pictorial description comparing the three species is shown in Figure 7. We analyze each class separately. Other researchers can view the monad map differently, depending on their area of interest, and herein lies the power of the ontological analysis in that it is a transparent classification scheme.

### 4.1. Metallic Silver

**Figure 8:**
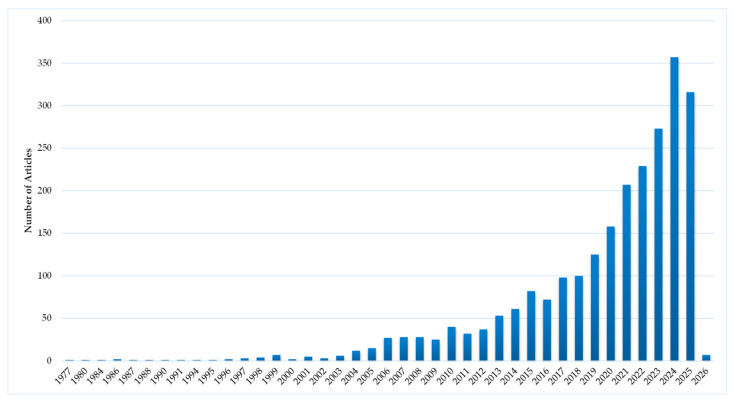
Publications with nanocrystalline silver.

Amongst the silver species, this area is the most extensively researched with 2425 articles (Figure 4). The temporal distribution of these publications is shown in Figure 8. There has been an exponential growth of publications in this area over the last three decades. This is primarily driven by research on AgNPs (metallic Ag particles with sizes less than 100 nm), with 91% of articles featuring this form of silver. Analysis of Figure 4 and Figure 7 suggests the following:

- AgNP are the main class of silver that is being investigated for wound dressings.

The interest in nanoparticles (NPs) is manifested because of their unique properties including quantum effects, surface effects, and reactivity including dissolution chemistry [58]. AgNPs are usually synthesized by the reduction of silver ions, and there are numerous chemical routes, including the use of inorganic reducing agents (such as NaBH_4_) [59], organic reducing agents (such as ascorbate) [60], and biological reducing agents (such as natural products) [61]. There is also an incredible diversity in the sizes and shapes of AgNPs [62]. These colloidal particles often need to be stabilized by surface-active agents that minimize the aggregation of the AgNPs [63,64,65,66,67]. The release of silver ions from AgNPs depends on the size (smaller sizes with higher surface areas are more prone to dissolution), shape and surface coating [68]. Because of the multifaceted nature of AgNPs both via different synthesis methods and physico-chemical characteristics, there is considerable basic research in this area [69].

- Most of the research with AgNPs focuses on evaluating their antimicrobial potency via in vitro tests with planktonic bacteria and determining cytotoxicity.

This is possibly done to establish the bona fide antimicrobial properties of AgNPs to reduce the bioburden and for comparison with previous studies or new synthetic methods. The methods of choice for potency are determining Minimum Inhibitory Concentration (MIC—the lowest concentration that prevents visible growth of bacteria) and Minimum Bactericidal Concentration (MBC—the lowest concentration that kills >99.9% of the initial bacterial population) using both Gram-negative and Gram-positive bacteria, with the most common bacterial species studied being *E. coli*, *A. baumanni*, *P. aeruginosa*, *S. epidermis* and methicillin-resistant *S. aureus* (MRSA) [70]. The cytocompatibility of AgNPs is also a topic of much research since cytotoxicity to mammalian cells can delay wound healing [71]. The most common method of measuring cytotoxicity is based on visible spectroscopy, and the ability of viable cells to reduce MTT (tetrazolium salt) to formazan (also mentioned as the XTT and WST-1 assays). The choice of viable cells is broad, including fibroblasts and keratinocytes. The adaptation of new structures, such as 3D-printed AgNP-assemblies for wound healing, will depend on their cytotoxic effects [72].

- A significant amount of research exists on silver dissolution from AgNPs in different media and as a function of surface ligands [73], with ICP-MS and visible spectroscopy being commonly used.
- Fewer studies focus on how the AgNPs dissolve and the time dependence of the process, which is crucial for applications in wound healing
- There is not much work on incorporating AgNPs onto other nanosupports; rather, AgNPs are used directly supported on various matrices. There are examples of the use of nanozeolites [74] and nano-MOFs [75]. The advantage of these nanosupports could be a more controlled release of silver.

Since the pioneering study by Winter [76] showing that moist wound epithelializes faster than dry wounds, much effort has been placed on moisture control via wound dressings. Also, in the case of AgNPs, the dressing matrix is critical, because contamination by wound exudates can compromise silver release [36]. Low humidity (<40%) dries the skin, whereas high humidity (>60%) risks infection [77]. The main supports used for AgNPs are hydrogels and chitosan. Hydrogels are chosen because they can maintain moisture in the wound environment, which is important for wound healing. Hydrogels are polymeric materials that are cross-linked physically or chemically. Physical crosslinking uses physical entanglement, hydrogen bonding, or Coulombic ionic interactions, whereas chemical cross-linking involves covalent bonds [78]. Hydrogels are 3D structures that are hydrophilic, flexible, and biocompatible, with controlled release characteristics and a resemblance to living tissues. Recent examples of hydrogels used with AgNPs include pluronic f-127 (19%) hydrogel [79], polyvinyl alcohol (PVA) hydrogels [80] and chitosan-polyethylene glycol (PEG) hydrogel [81]. The potential of AgNPs in various different matrices, including hydrogels, hydrofibers, gauzes and nanofibers for chronic wound healing has been recently reviewed [82]. Chitosan used with AgNPs provides added antimicrobial power and complements the AgNPs. The exploration of supports also stems from modifying AgNP dissolution kinetics. Natural polymers are biocompatible and are an attractive feature.

- For mechanistic studies of wound healing, studies on inflammation have been the focus with fewer studies on proliferation and remodeling. The inflammatory markers commonly examined are TNF- α, TGF-β, IL-1, IL-1 beta, IL-6, IL-4, IL-13. In healthy mice, AgNPs increased TNF-α, CXCL1, TGF-β, HO-1 and IL-4 expression within the mouse spleen [83]. AgNPs were found to upregulate the cytokine gene expression of IL-1, IL-6 and TNF-alpha in primary blood monocytes, indicating a potential for harmful effects [84]. AgNP were found to inhibit the production of proinflammatory cytokines and reduce the activation of NF-κB, suggesting a pathway to improve wound healing [85]. Similar effects were noted for a lung epithelial line, with AgNPs inhibiting the NF-κB transcriptional and inflammatory pathways [86]. Considering the large number of publications on AgNPs, studies on animals and humans are sparse. AgNPs dispersed in Carbopol gels demonstrated wound healing in incision, excision and burn models in Wistar rats, with minimal adverse toxic effects on the healing tissues [87]. Recent review articles highlight the role of AgNPs in different matrices on the healing observed in rat and rabbit wound models, along with events proceeding at the cellular level [88,89].
- A large fraction of the papers on AgNPs report dressing design and healing time. Considering that there are only a few papers on animal/human studies, our assessment is that this is because basic research papers claim these as potential advantages.

However, basic research is still the focus with AgNPs, and clinical translational research is yet to materialize [90], as is evident from the monad map.

### 4.2. Inorganic Silver Species

**Figure 9:**
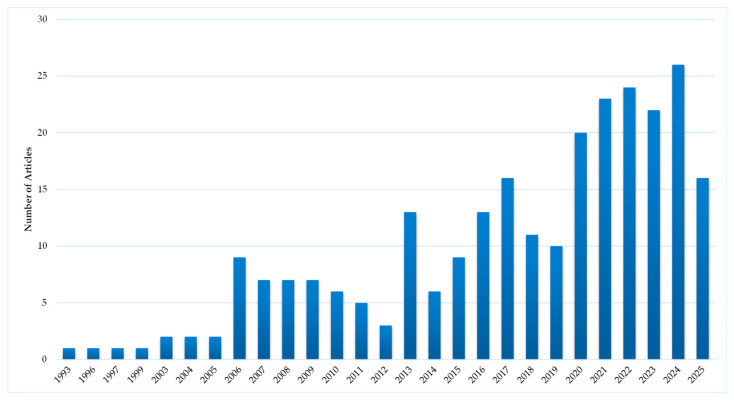
Publications with inorganic silver species.

Examining different inorganic compounds of silver ions does not seem to be an active area of research, as evidenced by the number of publications and their evolution over time, shown in Figure 9. This group also includes encapsulated silver ions, held in supports such as metal–organic frameworks, zeolites and zirconium phosphates [91].

The monad map shown in Figure 5 and Figure 7 indicates only 263 articles in this area. The most striking feature of the monad map is the relatively large number of human and animal studies. There are more human studies than in vitro studies. This is an indication that basic research is not the predominant focus of the research on inorganic silver species, whereas there is considerable clinical research. Why is this the case? A review of FDA-cleared wound dressings provides a possible answer. On the FDA database, there are 123 entries for silver-based wound dressings (Search performed at https://www.accessdata.fda.gov/scripts/cdrh/cfdocs/cfpmn/TextResults.cfm with keywords “silver wound dressing” on 15 March 2026). An analysis of these FDA-cleared products includes eight AgNPs (majority being the Acticoat family of dressings), 90 silver ion/metal, 18 silver organics, 7 miscellaneous compounds. Most of these dressings are based on inorganic silver species (73%), many of which are commercially available. We hypothesize that the commercial availability of FDA-cleared products has facilitated clinical studies.

Our group has recently published a detailed review focused on commercial silver wound dressings, with considerable details on their use in clinical studies [36]. The effectiveness of silver wound dressings in managing chronic wounds is still being debated. When wound healing is delayed due to comorbidities, the wound can become infected and persist in an inflammatory phase. Biofilms develop in these wounds, which are marked by increased exudate formation, malodor, and delayed wound healing, decreasing the patient’s quality of life. Analysis of 155 studies with 5046 patients suggested that silver dressings improved the healing rate and shortened healing time, but had no effect on wound surface area [92], a conclusion consistent with other studies: that the silver dressings have marginal therapeutic advantages in chronic wounds [37]. Another paper examined 59 studies, and concluded that the quality of clinical research with silver is poor and it may have benefits only for the first few days/weeks [93]. The cytotoxic effects of silver on keratinocytes and fibroblasts in delaying wound healing have not been examined in clinical studies [94]. There are few random-controlled trials (RCTs) with commercial dressings. With a dressing of silver ions dispersed in a polyurethane foam, an analysis of four RCTs studies suggested that the dressing had a beneficial effect on moderately to highly exudate wounds [95]. Finally, the ontology indicates that for this class of silver, healing time has been a major focus in wound recovery, though the current clinical focus has been shifting to the quality of life for the patient.

### 4.3. Organic Silver Species

**Figure 10:**
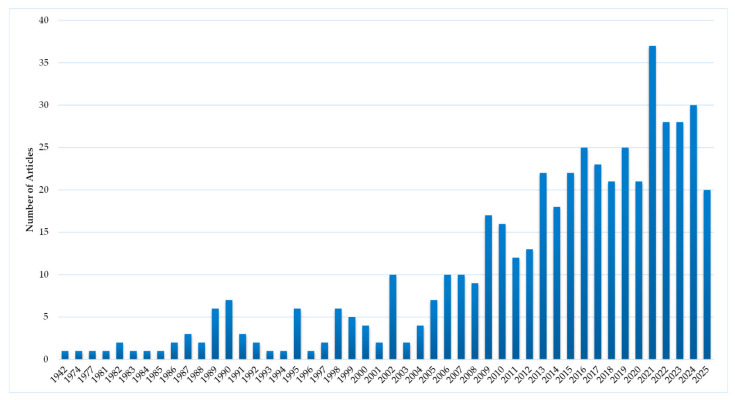
Publications with organic silver species.

This class of silver nets 492 articles and is dominated by silver sulfadiazine (Figure 6). There is a significant amount of work in humans and animals, (65% of papers) primarily with burn wounds. The focus has been on healing time. Figure 10 shows the number of publications in this area over time. The growth was linear in the early years, but it has tended to stabilize over the past five years. It is interesting to consider the reasons why there have been extensive clinical studies with silver organics, in particular silver sulfadiazine (SSD). Prior to the 1960s clinical treatment of burns involved silver nitrate solutions, which minimized bacterial growth but caused stains [96]. Fox’s formulation of SSD as a stable, water-insoluble salt that combined the antimicrobial action of silver with the sulfonamide effects of sulfadiazine was reported in 1968 [97], followed by a patent also in 1968, and FDA approved SSD for burn wound infections in 1973. Fox’s innovation soon became the gold standard for topical antimicrobials for burns. After the introduction of SSD therapy, mortality in burn populations dropped from 38 to 45% to 14–25% [98]. With burns, the body’s resistance to infectious bacteria is reduced. Healing time has been the focus of wound recovery, with pain being the secondary focus. The focus on pain is not surprising; early applications of SSD required frequent change, causing patients a lot of discomfort. SSD dressings with controlled release are now available [36]. Wound healing as measured by epithelization has been a focus.

Though scarring in wound healing is an important clinical indicator, not much research focuses on this topic across any of the silver species. Also lacking is an emphasis on cost, though this will be an important feature of the clinical use of silver wound dressings.

It does appear though that the use of SSD for burns is going to decrease. Though it is considered extremely safe, the American College of Surgeons/American Burn Association have recently recommended (2024–2026) avoiding SSD as first-line for partial-thickness burns. They suggests that petrolatum/triple-antibiotic ointments are preferable due to superior healing/infection control (https://praxismed.org/article/de3d65aa-57e6-4974-9083-ac9796b9adb2?z=0 (accessed 15 April 2026)). After decades of use of SSD, the data indicates that SSD increases the risk of infection, prolongs hospital stay and delays wound healing, and recommendation is that it should not be used when there is visible evidence of healing. The high initial release of silver ion (3176 ppm) upon application of SSD can cause toxicity. Even as early as 2012, an evaluation of RCTs showed that there was insufficient evidence to support or refute the use of SSD for partial-thickness burns [99]. A recent RCT analysis of comparison of AgNPs and SSD concluded that the nanocrystalline silver dressings were superior in improving wound healing time and reducing the frequency of painful dressing changes in burn patients, but the overall certainty of evidence was low to very low [100].

## 5. Discussion

**Figure 11:**
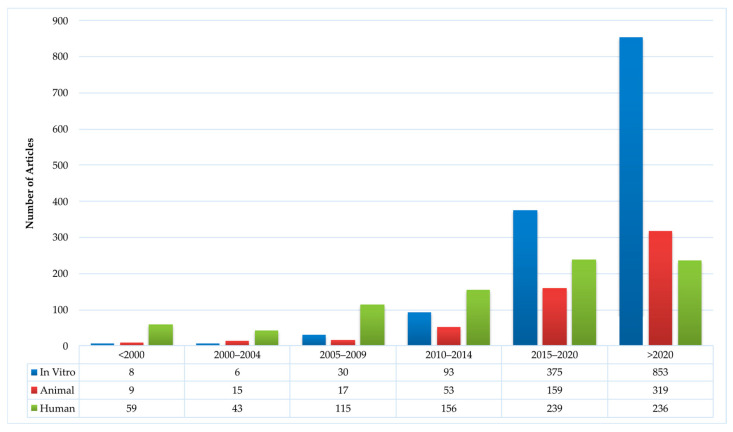
Distribution over time of papers on silver examining in vitro, animal and human tests.

As we survey the entire monad maps, there are certain issues that stand out and merit discussion. The overriding goal stated in the research papers, even the most basic ones, is to develop novel silver wound dressings. There is a certain progression typically for the evolution of medical products: basic research focused on improving potency, animal studies and clinical research. Figure 11 shows how in vitro, animal and human studies have evolved over the past three decades as determined from the ontology. The increase in animal and clinical studies over the past decade is a good indicator that in the future, there will be new silver products on the wound dressing market.

### 5.1. Road to Commercial Products

The ontology suggests that there are trends towards more commercial products. We now address some of the issues that need to be dealt with for a successful drive towards clinical applications. We frame this discussion based on a series of questions suggested by the ontology:

1. Why are there more human studies with the inorganic and organic silver species than AgNP?

**Figure 12:**
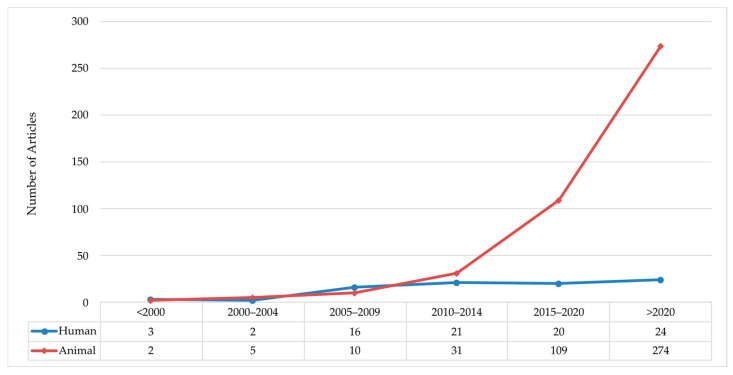
Distribution of papers over time of AgNP in animal and human tests.

We suggest that this is the case because there are few FDA-cleared products featuring AgNPs, as compared to the other two classes. Figure 12 presents the temporal distribution of AgNPs in animal and human studies. This is an encouraging trend, especially the large increase in animal studies, which suggests that the potential for novel effects in clinical studies with AgNPs may be forthcoming.

2. What will it take for the proof of concepts studies on AgNP (95% of papers) to translate to clinical success?

Translating abundant lab research on AgNPs into safe, reliable, cost-effective wound dressings is an uphill battle. There is increased regulatory scrutiny around NPs and their environmental impact. Furthermore, multiple technical, clinical, regulatory and commercial barriers will probably prevent most AgNP concepts from reaching the market.

In order to address these barriers, the following needs to be addressed:

- Controlled silver release and stability—The primary activity of AgNPs is thought to proceed via oxidative dissolution to Ag^+^; thus any surface modification will influence this activity. The biological milieu also contains numerous ionic species, such as chloride and sulfide, as well as proteins that can bind to the surface and influence antimicrobial activity. The size of the AgNP will also influence dissolution, smaller particles dissolving more rapidly. Shape of the NPs also influences antimicrobial activity, with nanoprisms > nanorods > nanospheres [101,102,103,104]. Effective antimicrobial action requires controlled ion release, this is difficult in a complex biological environment. Many AgNP constructs either release too much (toxic) or too little (ineffective). Nanoparticles also aggregate or change during storage/sterilization. Surface modification and surface charge, which determine aggregation, influence dissolution kinetics [105]. Much of the basic research on AgNPs correctly deals with these issues.
- Safety and toxicity uncertainties—There is some concern that AgNPs may function in additional ways than Ag^+^. Toxicity of Ag^+^ is manifested as oxidative stress, and alteration of cellular activity due to Ag^+^ complexation with important functional species. AgNPs can manifest their toxicity both as Ag^+^ and through additional routes, such as direct interaction with the bacterial membrane, leading to biomechanical changes, formation of pits as well as penetration of the toxic NPs into the cell. How this translates to the interaction of AgNPs with mammalian cells is an active area of research [106,107]. Concerns regarding long-term systemic or local toxicity of AgNPs complicate the regulatory approval process. There is also the issue of microbial resistance to AgNP [108,109]. Furthermore, argyria, even though primarily a cosmetic issue, remains a cause for concern [110].
- Reproducibility and standardization—The synthesis methods for AgNPs are extremely diverse. Scaling the synthesis process to produce reproducible, tightly controlled NP batches is nontrivial.
- Manufacturing and cost—Robust GMP manufacturing, including the purification of NPs, sterilization, packaging, and shelf-life stability for NP dressings adds complexity and cost compared to ionic silver or organic silver dressings. Moreover, there are concerns regarding AgNP toxicity via inhalation [111]; therefore the manufacturing process needs to address these concerns.
- Regulatory hurdles—Regulators require well-characterized materials, toxicology data, and clinical evidence. Nanomaterials raise extra data expectations (biopersistence, biodistribution) [22] and no harmonized global standards exist as of yet.
- Clinical evidence and market adoption—There is a need for to be higher-quality randomized trials showing clear superiority over current silver dressings. Without a clear clinical advantage, investors will hesitate.
- Environmental and disposal concerns—Concerns over the potential ecological impacts of NP release during manufacture and during use also raise further regulatory and procurement resistance. AgNPs can bind to aquatic organisms and alter the marine ecosystem.
- IP and commercialization—The patent landscape needs to be transparent. Licensing issues and a lack of industry partners to scale particular academic formulations will slow translation.
- Effective alternatives exist—Ionic silver, silver sulfadiazine, and advanced dressings already perform acceptably for many indications, thereby reducing the commercial incentive.
- There is always market resistance to experimenting with new products, unless the benefits are clear.

3. What areas of basic research are necessary to provide a breakthrough in the clinical applications of any silver-based dressing?

Chronic wounds take longer to heal (>3 months), the reason is that the normal phases of wound healing, hemostasis, inflammation, granulation, epithelization, contraction, and remodeling, are not functioning optimally. Such wounds exhibit biofilms, prolonged inflammation and tissue damage [112,113,114,115,116]. The wound environment is ideal for bacterial infection and biofilm development, especially for immunocompromised patients. The surface extracellular polymeric substance (EPS) matrix of a biofilm is difficult to penetrate. For silver alone to disrupt biofilms, high concentrations are required, which can be toxic. So, strategies for disrupting biofilms need to be incorporated into dressings. Several dressings use adjuncts such as surfactants to disrupt biofilms [117,118,119]. Another area of basic research is the investigation of polymicrobial biofilms. For human chronic wounds, *S. aureus* and *P. aeruginosa* are present, with the latter residing much deeper within the wound [113]. This brings up another area that is sparsely investigated. what are the spatial diffusion characteristics of the silver actives in the wound, and can they penetrate deeper into the complex three-dimensional wound topologies [119]? The reason that silver actives may not be able to penetrate deeper into wounds where bacterial colonization has taken place due to because of precipitation and deactivation in the biological milieu (wound exudate) [36]. Nanosupports may facilitate in deeper deliveries. The concentration of actives used is relevant for the deeper penetration, but higher concentrations can lead to toxicity.

4. What types of animal/human studies are needed?

Appropriate animal models that mimic chronic wounds are necessary; and there are several available, including ischemic wounds, ischemic reperfusion wounds, pressure ulcers, and diabetic wounds [120,121,122,123]. The most valuable human trials are randomized controlled trials, and they minimize bias. The focus should be on wound healing (wound area reduction and wound remodeling) versus just reducing bioburden. The opinion on the efficacy of silver dressings is mixed. The VULCAN study, focusing on randomized clinical studies, found no advantage for silver dressings in the treatment of venous ulcers [124]. International Working Group on the Diabetic Foot Ulcers also did not recommend silver dressings for routine ulcer management [125]. Another international group recommended that silver dressings can reduce bioburden and result in shorter hospital stays [126].

5. What is the competition?

There are currently other antimicrobial treatments that are alternatives to silver, including antibiotics, other metals (zinc, copper), natural antimicrobials (honey especially for burns, chitosan), and iodine compounds. New treatments on the horizon include antimicrobial biologics, specifically antimicrobial peptides and proteins, and the use of near-infrared radiation (photodynamic therapy) [127,128,129].

### 5.2. Advantages of the Ontological Method

The present ontological review differs from the traditional qualitative reviews, meta-analyses, systematic reviews, reviews of clinical studies, and bibliometric analysis in four fundamental ways, which are discussed below.

First, the ontological review is a top-down review based on a logically constructed theoretical framework of the problem. The other reviews are bottom-up reviews, grounded in published articles on the subject. The latter are subject to significant errors of omission; logical elements, segments, and pathways to address the problem that are not in the literature are unlikely to be discovered. On the other hand, the ontological review highlights these elements and dimensions that have received little or no attention in the research. For example, Figure 3 shows that several areas need more research: silver in other nanosupports such as zeolites and MOFs for controlled delivery of silver, diffusion of both the amount of silver in the surrounding medium and the depth of the penetration, comparisons to other antimicrobial agents, and research on scar tissue and the costs of the material, the latter being very important for clinical adoption.

Second, the ontological review is inclusive, based on the entire population of relevant articles on the subject. The other reviews (except bibliometric reviews) are selective, usually based on a sample of articles selected based on specific criteria such as the method, topic, quality, and sample. Consequently, there is no sampling bias in the results of the ontological review, and it describes the state of research regarding the entire problem rather than just a section of the problem. So, the ontology discussed here focused on all 4711 articles.

Third, the ontological review is based on a theory of the problem presented as an ontology. The other reviews are methods-based and not theory-based. As a theory, the ontology has the power to be used to (a) describe the elements, dimensions, and pathways to address the problem, (b) explain the dynamics of the pathways, (c) predict the outcomes of the pathways, and (d) regulate the pathways. The other reviews are atheoretical, and their results provide neither a systemic view of the problem nor systematic pathways to address it. This is evident in Figure 3, the monad map of the entire corpus of articles.

Fourth, the ontological analysis can be used to develop a systemic roadmap for systematic future research to address the problem. The other reviews often yield only local roadmaps and incremental directions for future research. The results highlight the dimensions, elements, and pathways (or segments) that have been heavily emphasized, lightly emphasized, and or not emphasized at all. The heavy emphasis may be because of the subject’s importance, ease of doing research, its long history, or a combination of the three. The light emphasis may be because of the subject’s unimportance, difficulty of doing research, its short history, or a combination of the three. The lack of emphasis may be because of a blindness to the subject’s importance, the impossibility of doing research, or a combination of the two. Based on the ontological mapping and the above analysis one can formulate a roadmap for research that (a) reinforces the pathways that are likely to be effective, (b) redirects the pathways that are likely to be ineffective, and (c) experiment with novel, innovative pathways (for example, combinations of silver species and nanosupports) that have not been explored. These pathways can be periodically updated, like a ‘Google Map’, based on feedback and learning from the published research. A decadal ontology map of the entire field can identify the direction of specific research over time, as demonstrated in Figure 11 for the in vitro, animal and human studies.

Overall, the ontology and the method of ontological analysis can complement traditional reviews in the field and add distinctive value to its advancement. Overall, the power of an ontology is that with one look one can see which research areas have enjoyed much popularity while others have not. In this review, we have pointed these issues and provided suggestions for research necessary, for example, if AgNPs were to be used in clinical wound dressings. Also, it is clear that dressings with SSD have probably enjoyed their popularity and are now on the decline, for good clinical reasons that we have outlines.

## 6. Conclusions

This review is the first attempt at making an ontological map of the research on antimicrobial silver for wound dressings. This issue is logically constructed as: the use of **silver** species on **nanosupports** deposited on a **matrix,** with **antimicrobial effectiveness** assayed by **methods** to promote **wound healing** of chronic **wounds** as determined by **recovery.** Each bolded term is a dimension of the ontology and denotes a taxonomy of constituent elements. Considering this globally, the ontology articulates 780 × 7 × 80 = 436,800 potential pathways to design silver-based wound dressings. An example of a potential pathway is: elemental nanocrystalline silver species in nano-MOFs deposited on a support with potency effectiveness tested in vitro to promote the healing of chronic vascular wounds as determined by the dressing. The ontology expresses the combinatorial complexity of the problem fully. An exhaustive search in the Scopus database led to the discovery of 4711 articles. We mapped the pathways (and segments of the same) that have been researched in 3077 of these peer-reviewed articles on the subject. A Convolutional Neural Network (CNN) was trained and then used to map the entire population of articles. With a focus on silver, we could separate the field into three classes: nanoparticles of silver (AgNPs), inorganic silver compounds and organic silver compounds. The majority of research (78% of articles) over the past decades has focused on AgNPs, and the number of papers is experiencing an exponential increase. Interestingly, even though there is an enormous focus on AgNPs, most human studies are done with inorganic and organic silver compounds. We hypothesize that this is because inorganic and organic compounds of silver cleared regulatory requirements early on and are in clinical use, making them accessible for human studies. However, we find that the number of papers on AgNPs in animal studies has been increasing over the past decade, indicating that there are potential clinical products in the future. Based on the ontology, we could also identify areas where more basic and applied research is needed. One such area is the extent of penetration of the silver actives into the complex 3D topology of the wound. We suggest that a decadal ontology map of the field will provide a clear picture of the evolution of the field, a sort of Google Map driven by a survey of the peer-reviewed research.

## Data Availability

The original data consisting of scholarly articles used in the study are available from Scopus (www.scopus.com, accessed on 29 April 2026).

## Supplementary Materials

The following supporting information can be downloaded at: https://www.mdpi.com/article/10.3390/antibiotics15050462/s1. The Supplementary Section of this paper explains the rationale of the choice of the elements of the taxonomy and details of the search method.
