# Supplementary material for: Research on Silver-Based Wound Dressing: An Ontological Analysis
Source: Antibiotics (Basel). 2026 May 2;15(5):462. doi: 10.3390/antibiotics15050462 (PMC13203702; doi:10.3390/antibiotics15050462)
Supplement: Supplementary file 1 [file antibiotics-15-00462-s001.zip › antibiotics-4228447-supplementary.pdf]

Supplementary Materials

Section S1. Justification of the taxonomy of the ontology.

Silver, in different forms, is an antimicrobial agent that can be used to treat chronic wounds. The antimicrobial effectiveness of a species of silver can be regulated by encasing it in a nanosupport and depositing it on a suitable matrix for delivery to the wound site. The silver species, nanosupport, and matrix combination must be designed to regulate the antimicrobial potency, the humidity of the wound environment, and the diffusion of the potency over time and at different wound depths. This logic is articulated in the first four dimensions (columns) of the ontology of silver-based nanosupport wound dressing and described next.

S1.1. Silver

The first dimension, Silver, denotes the species of silver that can be used in wound dressing because of their antimicrobial properties. The three broad species are elemental silver, inorganic silver compound, and organic silver complexes. The elemental silver species includes nano crystalline and metal ion forms; the inorganic silver compound species includes silver oxide, silver chloride, silver phosphate, silver sulfate, and silver-zinc, silver-copper, and silver-metal-ion combinations; and the organic silver complexes species includes silver sulfadiazine, silver alginate, and silver carboxymethyl cellulose. These silver species and their constituents denote a comprehensive, current taxonomy of silver forms that can be used in wound dressing because of their antimicrobial properties. Table S1 denotes the taxonomy of silver species.

Table S1. Taxonomy of silver species.

Elemental-Nanocrystalline	Well-defined long-range order of making up the material in its metallic form
Elemental-Metal Ion	Metallic ions, Ag^+ (same as Ag(I))
Inorganic-Silver Oxide	Ag_2O and other forms having Ag and O in their composition
Inorganic-Silver Chloride	AgCl, any forms having silver and chlorine
Inorganic-Silver Phosphate	Ag_3PO_4, any forms having Ag, P, and O
Inorganic-Silver Sulfate	Ag_2SO_4, any forms having Ag, S, and O
Inorganic-Silver, Zinc, Copper, Metal Ion	Can be any metallic ion, such as copper ion or zinc ion (Cu^{2+} or Zn^{2+}) in combination with silver
Organic- Silver Sulfadiazine	Well-defined chemical substance comprising silver ions and sulfadiazine
Organic- Silver Alginate	Complex of silver and alginate
Organic- Silver CMC	Silver incorporated in carboxymethyl cellulose

Another advantage of the taxonomy approach is that silver species can be extended by adding new species and their corresponding constituents.

S1.2. Nanosupport

The second dimension, Nanosupport, denotes the taxonomy of nanosupports currently used for delivering the silver species to the wound. It includes nanoparticles, nanozeolites, nanolipids/lipid nanoparticles, nano-metal organic frameworks (MOF), nanopolymers, and nanochitosan.

The six types of nanosupports in combination with the ten forms of silver denote sixty potential combinations of the two to deliver the antimicrobial agent to the wound.

Examples include: (a) elemental nano crystalline silver species in nanoparticles, (b) organic complex of silver sulfadiazine silver species in nanochitosan, and (c) inorganic compound of silver chloride silver species in nanopolymers. Table S2 denotes the taxonomy of nanosupport.

Table S2. Taxonomy of nanosupport.

Nanoparticles	Submicron particles
Nanozeolite	Submicron zeolites, silver is introduced via ion-exchange
Nanolipids/Lipid Nanoparticles	Includes liposomes, any particle made up of lipids and submicron in size
Nano Metal Organic Framework (MOF)	Metal organic framework such as ZIF, imidazolate and submicron in size, often written as zeolitic-like MOF
Nanopolymers	Polymers with any dimensions in submicrons, such as electrospun fibers, silver is incorporated during the electrospinning process
Nanochitosan	Chitosan particles of submicron size

The taxonomy of nanosupport can be extended in the future with the addition of new ones.

S1.3. Matrix

The third dimension, Matrix, denotes the substrates that can be used to deliver a silver species and nanosupport combination. There are thirteen types of substrate matrices. The list includes support, natural polymers, hydrogel, collagen/cellulose, alginates, foams, gels, starch, hydrofiber, silk fibroin, hyaluronic acid, chitosan, and synthetic polymers. This taxonomy too can be extended in the future with the addition of new substrates. Table S3 denotes the taxonomy of matrix.

Table S3. Taxonomy of matrix.

Support	Any reported material that has the potential to be used as a matrix for wound dressing, whose potential has still not been demonstrated in animals or human studies, also includes scaffolds
Natural Polymers	Any polymer that has building blocks found naturally, such as amino acids, sugars etc.
Hydrogel	3-D network of hydrophilic polymers that retain water
Collagen/Cellulose	Also, a natural polymer
Alginates	Anionic polysaccharide polymers from seaweed
Foams	A porous, low-density polymeric material
Gels	A semi-solid in which liquid phase is immobilized
Starch	Also, a natural polymer
Hydrofiber	Highly absorbent material that can gel
Silk Fibroin	Any form of silk, also a natural polymer
Hyaluronic Acid	A natural glycosaminoglycan polymer
Chitosan	A cationic chitin derived polysaccharide
Synthetic Polymer	Polymer synthesized with chemical components not naturally occurring, e.g., polyurethane

In combination, the first three dimensions denote 780 potential combinations of silver species, nanosupport, and matrix for wound dressing. Examples include: (a) elemental nano crystalline silver species in nanoparticles deposited on support, (b) organic complex of silver sulfadiazine silver species in nanochitosan deposited on silk fibroin, and (c) inorganic compound of silver chloride silver species in nanopolymers deposited on gels.

The taxonomy of support can be extended in the future with the addition of new ones.

S1.4. Antimicrobial Effectiveness

The fourth, Antimicrobial Effectiveness, dimension denotes the five attributes required in a wound dressing – potency, humidity, cytotoxicity, temporal diffusion (release of actives as a function of time), and spatial diffusion (depth of penetration of released actives into the surrounding medium). The 780 combinations discussed above must be assessed for their antimicrobial effectiveness on these five attributes to be used in wound dressing. Thus, a total of 780 x 5 = 3,900 pathways must be assessed to determine the effectiveness of silver-based nanosupport wound dressing. Examples include: (a) elemental nano crystalline silver species in nanoparticles deposited on support's potency, (b) organic complex of silver sulfadiazine silver species in nanochitosan deposited on silk fibroin's humidity regulation, and (c) inorganic compound of silver chloride silver species in nanopolymers deposited on gels and their cytotoxicity. It would be daunting to empirically list all the 3,900 combinations. One must (a) select the most likely combinations based on prior knowledge about the silver species, nanosupports, and matrices, (b) reject the unlikely combinations, and (c) delete the infeasible ones. Table S4 denotes the taxonomy of antimicrobial effectiveness.

Table S4. Taxonomy of antimicrobial effectiveness.

Potency	Antipathogenic activity towards any pathogens, including bacteria, viruses, fungi
Humidity	Mention of water adsorption, release and retention
Cytotoxicity	Toxicity towards living cells, also cytocompatibility and biological toxicity
Temporal	Release of actives from a material as a function of time, often mentioned as some concentration released into the surrounding environment
Spatial	Depth of penetration of actives into a biological milieu, how far are the actives penetrating

The taxonomy of antimicrobial effectiveness can be extended in the future with new attributes.

S1.5. Method

The arguments for the 80 wound healing and recovery properties of the 780 combinations of silver species, nanosupport, and matrix may be based on: (a) empirical evidence from in-vitro, animal, and human tests, (b) extension of the results from similar cases based on comparison, extrapolation, and generalization, and (c) analogy to other cases. These methods are denoted by the Method dimension of the ontology. Table S5 denotes the taxonomy of method.

Table S5. Taxonomy of method.

In Vitro	Study of cellular species, like different bacteria
Animal	In vivo studies on animals, birds, wound models are developed and studied on rats, pigs, and rabbits
Human	Explicit study of human subjects (in vitro and prediction that this will apply to humans not considered, coded differently)
Extension - Comparison	Any comparison of two or more materials related to any biological aspect of wounds
Extension - Extrapolation	Extrapolation of results to another system/material
Extension - Generalization	Generalizing observations to more subjects/materials than the focus of the study
Extension - Analogy	Making judgments based on results of a system to another system

The taxonomy of methods can be extended in the future to include other methods.

S1.6. Wound Healing

To promote effective healing, the dressing must promote hemostasis, be anti-inflammatory, limit wound proliferation, and stimulate tissue remodeling. These requirements constitute the sixth dimension, Wound Healing, of the ontology. Table S6 denotes the taxonomy of wound healing.

Table S6. Taxonomy of wound healing.

Hemostasis	Mention/discussion of clotting
Inflammation	Mention/discussion of studying inflammation, including inflammatory markers such as TNF
Proliferation	Mention/discussion of studying cell proliferation
Remodeling	Mention/discussion of studying tissue remodeling, including angiogenesis

The taxonomy of wound healing can be extended in the future to include other requirements.

S1.7. Wound

The seventh dimension, Wound, denotes the four common types of chronic wounds. They are vascular, diabetic, pressure ulcer, and burn wounds. These wounds require special care compared to other common wounds. Thus, the taxonomy of Wound denotes the following chronic wounds that take more than three months to heal:

- Vascular wounds: wounds perpetuated by impaired blood supply
- Diabetic wounds: wounds in a diabetic person, such as diabetic foot ulcers
- Pressure ulcer wounds: wounds arising from pressure or friction
- Burn wounds: injury caused by thermal, chemical, electrical or radiation

The taxonomy of wounds can be extended in the future to other chronic wounds.

S1.8. Recovery

Further, the healing of a wound must result in Recovery, as determined by the ease of dressing, pain management, healing time, scar tissue formation, and the cost, denoted by the last (eighth) dimension of the ontology. Thus, the object of silver-based nanosupport wound dressing is denoted by the 80 combinations of Wound Healing x Wound x Recovery. It includes promotion of: (a) hemostasis of chronic vascular wounds as determined by dressing, (b) inflammation of chronic diabetic wounds as determined by healing

time, and (c) remodeling of chronic burn wounds as determined by scar tissue. Table S7 denotes the taxonomy of recovery.

Table S7. Taxonomy of recovery.

Dressing Application	Tested on animal/human
Pain	Mention of pain in any form
Healing Time	Mention of healing in any form
Scar Tissue	Mention of scar in any form
Cost	Mention of cost in any form

The taxonomy of recovery can be extended in the future to include other requirements.

Section S2. Details of the Methodology

S2.1 The Preliminary Search

The preliminary search was conducted on Scopus in July 2025. The search strategy was guided by the ontology; individual parts of the search terms were derived from relevant taxonomies of the ontology. The preliminary search primarily focused on the following search terms in title, abstract, and keywords of articles: 1) silver, 2) nano*, 3) substrate OR dressing OR scaffold OR material, 4) wound OR infect*, and 5) healing OR care. The search process is denoted in the PRISMA diagram in Figure S1.

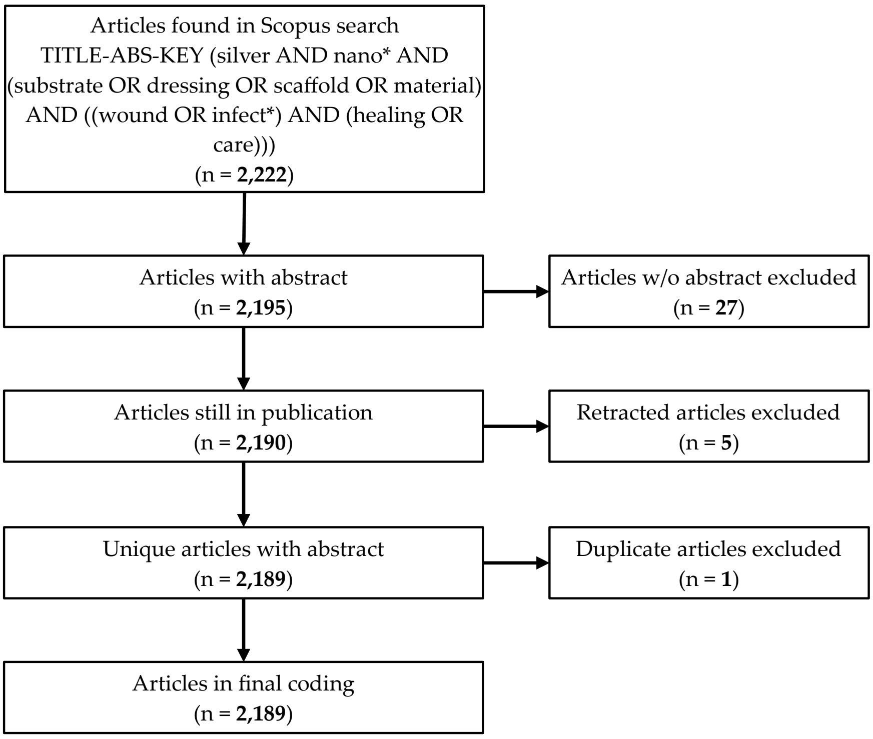

Figure S1. PRISMA diagram of preliminary search.

The preliminary search returned 2,189 articles after removing duplicate and retracted articles as well as articles without abstract. The corpus of articles was subsequently mapped to ontological elements.

S2.2 Mapping of Articles to Ontology

A random sample of 120 articles from the corpus was selected and manually mapped to the ontology by the coauthors. The sample was divided into 3 trenches of 40 articles, and each trench was coded by two coders using the taxonomical definitions described

above. Each coder coded 80 articles from two trenches and reconciled to produce a consensus coding set. The coding was recorded in a spreadsheet with lowest level ontological elements arranged in columns together with title, abstract, and keywords of individual articles. The coding is binary indicating the presence/absence of ontological elements in an article, 1 for presence and 0 or blank for absence. This coding set, designated as P0, was used as the initial training for mapping of the remaining articles in the corpus.

The mapping process followed a semi-supervised learning approach beginning with the dataset P0 containing manual coding of 120 articles. The P0 coding was divided into 10 folds of 12 articles each and validated following k-fold validation, a commonly used cross validation technique in machine learning. Ten CNN models were trained and tested. The CNN model of the best performing fold was used to generate coding of another set of 120 articles from the corpus. The coding set, designated as P1, was reviewed, revised, and reconciled by the coauthors. The combined coding of 240 articles from P0 and P1 datasets went through another k-fold validation with 10 folds. The best performing model was applied to coding the entire corpus of 2,189 articles constituting the P2 coding. The coding process is detailed in Figure 2.

S2.3 The Final Search

The final search for articles was performed in November 2025 after reviewing P2 coding. During the review, some articles on silver-based nanosupport wound dressing were missing in the coding, hence, there was a need to expand the corpus. Several trial searches with variations of the initial search terms revealed that removing "nano*" would expand the corpus to relevant articles while retaining those articles that were already captured in the initial search. The final search process is depicted in Figure S2.

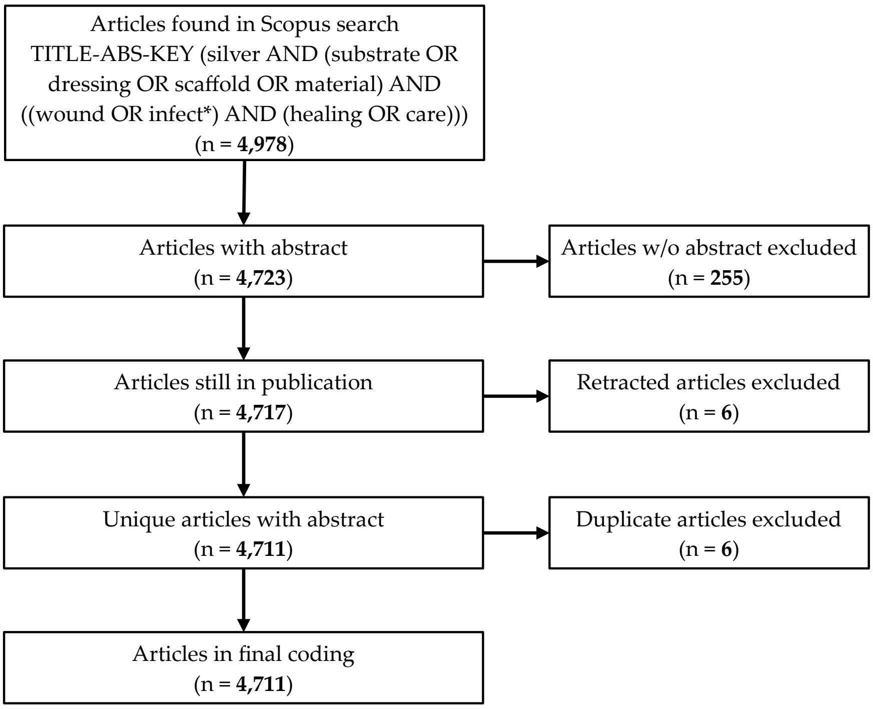

Figure S2. PRISMA diagram for final search.

The final search returned 4,711 articles. A random sample of 120 articles which did not appear in the initial search was selected and coded independently by the coauthors. Three sets of coding were integrated using the majority rule to generate the consensus coding designated as P3. The combined coding of P0, P1, and P3 was used to generate coding for all articles in the final corpus, the P4 coding. A quick review of the coding revealed that many relevant articles with "silver" in title and abstract were not coded. To

improve the accuracy of coding, a sample of 120 articles was randomly selected from that subset of articles and coded by the coauthors. The consensus coding after reconciliation was designated as P5 and subsequently used together with coding of P0, P1, and P3 to perform another round of k-fold validation and CNN-based coding of the entire corpus, P6. The final review of the coding by the coauthor with extensive knowledge of subject matter revealed that the coding was underrepresented in Silver Phosphate and Silver Sulfate elements. Six articles with these silver species specified in title and abstract were selected and coded as P7 coding. The final coding of all 4,711 articles in the corpus was generated by the CNN model trained and tested with the combined coding of 486 articles in P0, P1, P3, P5, and P7. The performance measures in Table S8 were computed for the coding of these 486 articles to benchmark the CNN model. The measures indicated that the CNN model achieved a very high performance replicating the coding provided in the training and testing datasets.

Table S8. Performance measures.

Accuracy	99.6%
False Omission Rate	0.3%
False Discovery Rate	1.6%
False Positive Rate/Fall-Out	0.1%
False Negative Rate/Miss Rate	3.7%
Negative Predictive Value	99.7%
Positive Preditive Value/Precision	98.4%
True Negative Rate/Specificity	99.9%
True Positive Rate/Sensitivity, Recall, Hit Rate	96.3%
Matthews Correlation Coefficient, phi	97.1%
Informedness, Bookmaker Informedness	96.2%
Markedness, deltaP	98.1%
F1 Score	97.3%
